# Detection of non-ST-elevation myocardial infarction and unstable angina in the acute setting: meta-analysis of diagnostic performance of multi-detector computed tomographic angiography

**DOI:** 10.1186/1471-2261-7-39

**Published:** 2007-12-19

**Authors:** Piet K Vanhoenacker, Isabel Decramer, Olivier Bladt, Giovanna Sarno, Charlotte Bevernage, William Wijns

**Affiliations:** 1 Department of Radiology and Medical Imaging, Onze Lieve Vrouw Ziekenhuis, Aalst, Belgium; 2 Cardiovascular Center Aalst, Aalst, Belgium

## Abstract

**Background:**

Multi-detector computed tomography angiography (MDCTA) has been increasingly used in the evaluation of the coronary arteries. The purpose of this study was to review the literature on the diagnostic performance of MDCTA in the acute setting, for the detection of non-ST-elevation myocardial infarction (NSTEMI) and unstable angina pectoris (UAP).

**Methods:**

A Pubmed and manual search of the literature published between January 2000 and June 2007 was performed. Studies were included that compared MDCTA with clinical outcome and/or CA in patients with acute chest pain, presenting at the emergency department. More specifically, studies that only included patients with initially negative cardiac enzymes suspected of having NSTEMI or UAP were included. Summary estimates of diagnostic odds ratio (DOR), sensitivity and specificity, negative (NLR) and positive likelihood ratio (PLR) were calculated on a patient basis. Random-effects models and summary receiver operating curve (SROC) analysis were used to assess the diagnostic performance of MDCTA with 4 detectors or more. The proportion of non assessable scans (NAP) on MDCTA was also evaluated. In addition, the influence of study characteristics of each study on diagnostic performance and NAP was investigated with multivariable logistic regression.

**Results:**

Nine studies totalling 566 patients, were included in the meta-analysis: one randomised trial and eight prospective cohort studies. Five studies on 64-detector MDCTA and 4 studies on MDCTA with less than 64 detectors were included (32 detectors n = 1, 16 detectors n = 2, 16 and 4 detectors n = 1). Pooled DOR was 131.81 (95%CI, 50.90–341.31). The pooled sensitivity and specificity were 0.95 (95%CI, 0.90–0.98) and 0.90 (95%CI, 0.87–0.93). The pooled NLR and PLR were 0.12 (95%CI, 0.06–0.21) and 8,60 (95%CI, 5.03–14,69).

The results of the logistic regressions showed that none of the investigated variables had influence on the diagnostic performance or NAP

**Conclusion:**

MDCTA of the coronary arteries performs good to excellent in the diagnosis of coronary artery disease in the acute setting and it can be used for early exclusion of NSTEMI or UAP in patients in the emergency department.

## Background

Acute chest pain accounts for approximately 6.5% of all emergency department visits in the US [1,2]. Failure to diagnose myocardial ischemia as a cause of acute chest pain has serious implications and the triage of patients with possible ischemia is often difficult. To reduce diagnostic error, many patients that present at the emergency department are admitted for observation, even when no initial ECG changes or elevated cardiac enzymes are present. Emergency departments have therefore developed chest pain units and diagnostic protocols commonly including serial cardiac enzyme evaluations and ECG's, supplemented with some form of stress testing with or without imaging [3]. Many of these patients are found to have no acute coronary syndrome (ACS) and more than 2 million patients with acute chest pain are admitted to the hospital without developing an ACS [4,5]. Data from Germany reveal that the number of potentially unnecessary hospital days is high, amounting to as much as 839 per 100 patients admitted for acute chest pain [6].

Non invasive access to coronary anatomy has become available with the emergence of multi-detector computed tomography (MDCTA) of the coronary arteries. Diagnostic performance of MDCTA has been evaluated in many studies [7]. Even though appropriate indications for MDCTA remain largely work in progress, the technique has been used as a tool to rule out ACS in the emergency department. [8-16]

The purpose of our study was to review the literature and perform a meta-analysis on the diagnostic performance of multi-detector computed tomography angiography for the exclusion of ACS in the emergency department. More specifically, we focused on the early non-invasive diagnosis of non ST elevation myocardial infarction (NSTEMI) with initially negative biomarkers and unstable angina pectoris (UAP). A second aim was to investigate the influence of multiple independent study-related variables on the diagnostic performance of MDCTA.

## Methods

### Study selection

A search in the Computer Retrieval of Information on Scientific Projects (CRISP), Database of Abstracts of Reviews of Effects (DARE), International Network of Agencies for Health Technology Assessment (INAHTA) and Cochrane Database of Systematic Reviews (CDSR) databases was done from January 1998 to June 2007. The purpose was to reveal the existence of structured reviews on MDCTA for ruling out ACS in the emergency department. A structured review or systematic review is based on a thorough review of the literature concerning a single topic but differs from a narrative review by statistically combining the results of several studies into a single outcome measure, by using techniques of meta-analysis. To search for original articles, a structured search of the PUBMED database from January 1998 to April 2007 was performed using an elaborated form of a previously described search strategy [17], by two authors (PV, OB). A second search was done by the same authors with another method recently described [18]. Both strategies can be found in Additional file 1. Reference lists of review articles and cited articles were used to locate additional studies. The following journals were hand-searched from January 1998 to March 2007: European Radiology, Radiology, Radiographics, American Journal of Roentgenology, Journal of Computer Assisted Tomography, Journal de Radiologie, Heart, The Lancet, New England Journal of medicine, JAMA, Journal of the American College of Cardiology, American Journal of Cardiology, American Heart Journal, Circulation, Hypertension, Circulation Research, European Heart Journal, British Medical Journal, Journal of Nuclear Cardiology, Emergency Medicine English Dutch, French, German, Italian and Spanish articles were also potentially included because the authors were familiar with these languages. Finally the results of all the searches were fused with bibliography software and duplicate publications were automatically removed (Reference Manager Professional edition version 10, ISI research software).

Studies were included in the meta-analysis if they met the following inclusion criteria: the data were acquired with a multi-detector CT-scanner with at least four detectors; catheter angiography was used as the reference standard and/or clinical follow up was obtained in all patients concerning the presence of UAP or NSTEMI ; the criteria for a positive result of MDCTA and CA were explicitly defined as 50 percent or greater diameter stenosis; the criteria for a positive clinical outcome (diagnosis of NSTEMI or UAP) where coherent with actual clinical standards and were explicitly defined; the absolute numbers of true-positive, false-negative, false-positive, and true-negative test results were available or could be derived from the available data or from the authors. These absolute numbers were accepted if they were derived on a per segment basis, or on a per patient basis. For segmental analysis an adapted American Heart Association 15 segment scheme of the coronary tree was used [19].

Exclusion criteria were: not an acute ACS setting or MDCTA later than 24 hours after the onset of the acute event; unknown status of cardiac biomarkers or positive markers; review article; not all patients were tested with the reference test (Table 1).

**Table 1:** Number and reasons for exclusion of articles that were reviewed in full-text.

Not an acute ACS setting	71
Unknown status of/or positive biomarkers	6
Review article	6
No comparison with reference standard in all cases	1
Total	84

The results of this search were analysed by two independent radiologists (PV, OB) as follows: Each investigator independently evaluated the retrieved studies for possible inclusion. In the case of conflicting findings as to whether a paper should be included, a decision was reached by consensus. In a first round articles were eliminated that clearly did not match the inclusion criteria, on the basis of the title or the abstract. In a second round, hard copies of the papers that gave rise to doubt on the basis of their abstracts were obtained and the full text was read, again eliminating a group of papers. The final group consisted of the included papers.

**Figure 1:**
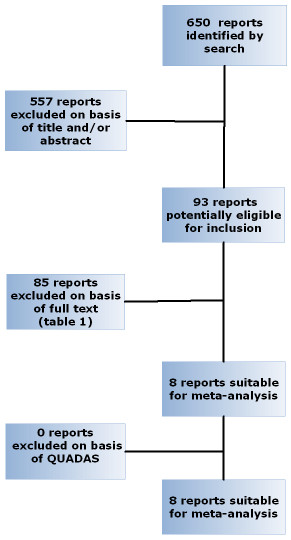
Flow diagram of the reviewing process.

Inclusion of studies was guided by the quality of the study design and report. A formal system for quality evaluation, Quality Assessment of Diagnostic Accuracy Studies (QUADAS,20), with a maximum of 14 points was used for judging quality in the final evaluation of included papers (Fig. 1, round 3). A score of >/= 12 was considered acceptable.

### Data extraction

The study parameters where extracted first independently and subsequently by consensus if a disagreement existed between the observers concerning the numeric value of a parameter (PV, ID). Data were extracted from the original articles taking into account the Standards for Reporting of Diagnostic Accuracy (STARD) checklist [21].

The absolute numbers of FN, FP, TP, TN were retrieved, calculated or requested from the authors. The numbers were calculated with Bayes theorem if only values for sensitivity, specificity, and predictive values were reported. This was done separately for per patient and per segment data, if available.

MDCTA was considered true-positive per patient, or per segment if at least one significant stenosis (>= 50% diameter stenosis) was found on MDCTA in, respectively, the investigated patient, or segment and confirmed on CA. MDCTA was considered true-negative if significant stenoses were correctly ruled out. MDCTA was considered false-negative if no significant stenoses were found on MDCTA and CA revealed at least one significant stenosis. MDCTA was considered false-positive if it revealed at least one stenotic stenosis and CA showed no significant stenoses. If no CA was done, positive clinical outcome of UAP or NSTEMI was the positive reference identifier, and absence of UAP or NSTEMI was the negative reference identifier with an analogue classification scheme as above.

Additional study characteristics were extracted (Table 2).

**Table 2:** Study Characteristics

	Year	Type	Det	Age	M/F	Prob	INC	NAP	Outc	NCA
White^8^	2005	PC	16	51	1.03	All**	69	0	CLIN	-
Gallagher^9^	2006	PC	64	49	0.53	Low	96	0.07	CLIN	5
Hoffmann^10^	2006	PC	16/64	57	0.53	All**	40	0	CLIN	-
Hoffmann^11^	2006	PC	64	54	0.58	All**	106	0.03	CLIN	8
Olivetti^12^	2006	PC	16	59	0.61	U	31	0	CA	31
Sato^13^	2006	PC	4/16	60	0.85	U	34	0.09	CLIN	-
Goldstein^14^	2007	RCT	64	48	0.43	Low	99	0.11	CLIN	12
Meijboom^15^	2007	PC	64	59	0,76	Low	33	0	CA	33*
Rubinshtein^16^	2007	PC	64	56	1.76	Int	58	0	CLIN	17

Type: Study type.

RCT: Randomized controlled trial.

PC: Prospective cohort.

Det: Number of detectors.

M/F: Male/Female ratio.

Prob: Pre-test probability.

INC: Number of included patients.

NAP: Non assessable proportion of patients = Ratio of non-diagnostic versus included patients.

Outc: reference standard used.

NCA: Number of patients that underwent Conventional Angiography.

Clin: Clinical.

U: Unknown.

*:only a fraction of the patients were included: the patients that clearly had negative biomarkers and were categorized as "low risk" in this study.

**: included patients with pre-test probability ranging from low to high

### Data synthesis and statistical analysis

Results are expressed as mean with 95% confidence intervals (95%CI), unless otherwise specified.

Interobserver agreement for study selection was evaluated with Cohen's Kappa test in which a value higher than 0.8 is considered to imply very good to excellent agreement. The three rounds of selection were evaluated.

The main analysis was performed at the patient level, as most studies focused on this level of information. We evaluated potential heterogeneity and inconsistency between publications [22,23] expressed with the Higgins and Thompson index which calculates the I^2 ^statistic, and is a derivative of Cochran's Q [24-26]. Cochran's Q displays a low power for detection of inconsistency when the number of studies is low, and a too high power when the number of studies is high.

Publication bias was assessed according to the method introduced by Sterne and Egger [27,28]. The existence of publication bias is expressed as an intercept value, and is 0 if no publication bias is found. A Funnel plot for graphic analysis of publication bias was constructed. A funnel plot is a plot of some measure of each study's sample size, such as the standard error, as function of its effect size. A distribution of the datapoints as an inverted funnel indicates that publication bias is highly unlikely. Calculations were performed with Statsdirect (StatsDirect Ltd. StatsDirect statistical software, England).

Summary estimates for sensitivity, specificity, positive likelihood ratio (PLR), negative likelihood ratio (NLR) and the overall diagnostic performance expressed in the diagnostic odds ratio (DOR) were calculated. This was done with a random effects model, which takes into account the variability between studies [29].

Subsequently, we performed a random effects SROC analysis to estimate the relationship between sensitivity and specificity, taking into account potential differences in positivity criterion (that is, the threshold used to mark a test as positive) and other factors of heterogeneity between settings. In a SROC analysis the logits (log odds) of sensitivity and 1-specificity are summed to calculate D, the log of the diagnostic odds ratio, and the logits are subtracted to calculate S, a proxy for the positivity criterion of the diagnostic test [30-32]. Then, a linear regression model D = a + bS is estimated, weighted by the inverse of the variance of D. Values for area under the curve (AUC) and Q* were calculated with their respective standard error (SE). Q* is the point of intersection of the SROC curve where SE and SP are equal and is a statistic that describes global diagnostic performance [33].

A multivariable logistic meta-regression was performed to investigate the influence of multiple explanatory variables on the diagnostic performance.

The evaluated variables included: Male/female ratio, mean age of patients, number of included patients, outcome used (CA or clinical), non-assessable patients excluded before analysis of diagnostic performance coded as a dichotomous variable (yes/no), number of detectors in the scanner used and pre-test probability of the cohort (low, intermediate, high, all or unknown) as indicated by the authors. Other variables such as scan and technique related variables were not investigated since in a prior meta-analysis these variables did not show any important influence [34] or could not be obtained in detail (such as coronary calcium score or pre-test probability). Variables with a significance level of p <= 0.10 were added to the multivariable meta-regression model in a stepwise forward manner. A variable was kept in the model if p < 0.05. A p <= 0.10 was used to add variables to the multivariable model whereas a p <= 0.05 was used to retain variables in the model. For adding variables to the model a higher p-value was chosen so as to increase the power of finding important effects. The beta-coefficients and corresponding relative diagnostic odds ratios from the meta-regression analysis indicated the effect of each variable on the overall diagnostic performance.

**Figure 2:**
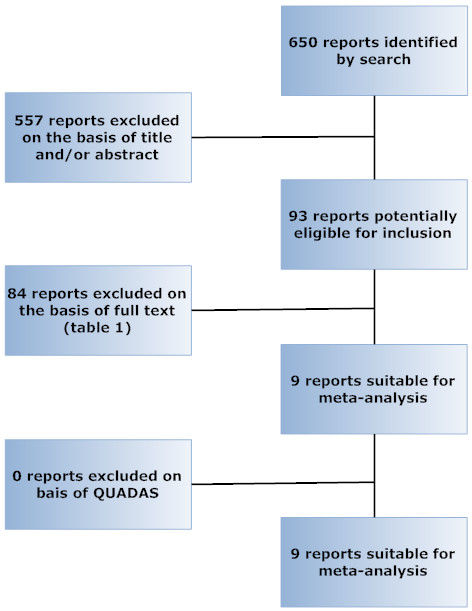
Flowdiagram of patient inclusion with patient categories used in summarizing data. In the studies analysed, from the eligible patients only a part was enrolled in the studies. The patients that were finally included was the subset of enrolled patients that completed the full protocol and that had diagnostic scans. Non assessable proportion (NAP) was the ratio of non-diagnostic patients or technical failures to the finally included patient.

The proportion of non-assessable patients (NAP) was pooled with a random effects model and evaluated as dependent variable in a multivariable regression analysis to evaluate which variables influenced this proportion. NAP was defined as the ratio of technical failure or non-diagnostic scans to the final number of included patients (Fig 2). The same variables except for the exclusion of non-assessable segments were tested as above. Software used for the calculation of pooled estimates, SROC analyses and all regressions was Meta-DiSc (version 1.3 Clinical Biostatistics Unit-Hospital Ramon y Cajal, Madrid, Spain) and/or Statsdirect

## Results

### Study selection and data extraction

The search in the CRISP, DARE, INAHTA and CDSR databases revealed no structured reviews or meta-analyses on the diagnostic performance of MDCTA for the assessment of NSTEMI or UAP in the acute setting.

The Pub-med search and the manual search for original articles resulted in 650 articles. 557 articles were excluded on the basis of their title or abstract, with 93 remaining for further evaluation. From these 93 articles, 9 where finally included in the meta-analysis [8-16]. Reasons of exclusion and numbers of 84 of these 93 studies are tabulated in Table 1. Five studies on 64-detector MDCTA and 4 studies on MDCTA with less than 64 detectors were included (32 detectors n = 1, 16 detectors n = 2, 16 and 4 detectors n = 1). One study was partially included (Table 2) since only a clearly defined fraction of the study patients were patients with negative biomarkers [15]. A flow diagram of the review process is given in Figure 1. In Additional file 2 the studies that were excluded were cited and classified according to Table 1.

Inter-observer agreement for the selection of articles between the two readers was 0.84, 0.87 and 1.0 for the first to third round respectively.(Cohen's Kappa).

Important study characteristics are displayed in Table 2.

All selected studies had a Quadas score of 14.

### Data synthesis and statistical analysis

A total of 566 patients where analysed. Only one study supplied information at the segment level, so that pooled analysis was only undertaken at the patient level.

Heterogeneity was not present among the studies when calculating the pooled DOR and NLR (I^2^, 0%), but was considerable for SE, SP and PLR (I^2^, 43.0%; 68.7%; 62.2% respectively), which justifies our choice of a random effects model.

**Figure 3:**
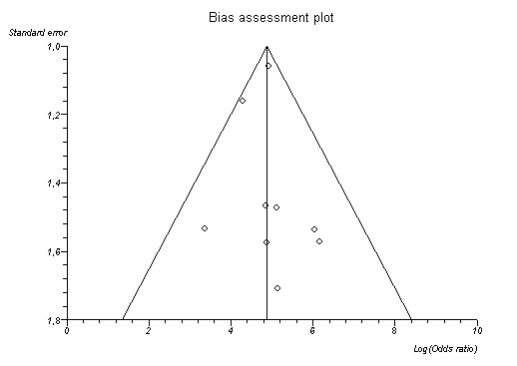
Graphical representation of publication bias. The dots, each representing one study are conforming to a triangular form, meaning that publication bias is low.

Publication bias was not present. (Intercept 0.89; p = 0.49); A funnel plot of the studies included is given in Figure 3. It shows a good conformation to the ideal, funnel shaped distribution.

Raw data and pooled sensitivity, and specificity are summarized in Table 3.

**Table 3:** Raw data from all the studies.

Study	TP/(TP+FN)	FP/(TN+FP)	Sensitivity (95% CI)			Specificity (95% CI)		
White^8^	10/12	55/57	0.83	0.52	- 0.98	0.96	0.88	- 1.00
Gallagher^9^	6/7	72/78	0.86	0.42	- 1.00	0.92	0.840	- 0.97
Hoffmann^10^	5/5	26/35	1.00	0.48	- 1.00	0.74	0.57	- 0.87
Hoffmann^11^	14/14	73/89	1.00	0.77	1.00	0.82	0.73	- 0.89
Olivetti^12^	15/18	13/13	0.83	0.59	- 0.96	1.000	0.75	- 1.00
Sato^13^	21/22	8/12	0.95	0.77	- 1.00	0.89	0.52	- 0.98
Goldstein^14^	8/8	88/91	1.000	0.63	- 1.00	0.97	0.91	- 0.94
Meijboom^15^	28/28	4/5	1.000	0.88	- 1.00	0.80	0.28	- 0.96
Rubinshtein^16^	20/20	35/38	1.000	0.83	- 1.00	0.92	0.79	- 0.98

TP: True positive.

TN: True negative.

FP: False positive.

FN: False negative.

95%CI: 95% Confidence intervals.

**Figure 4:**
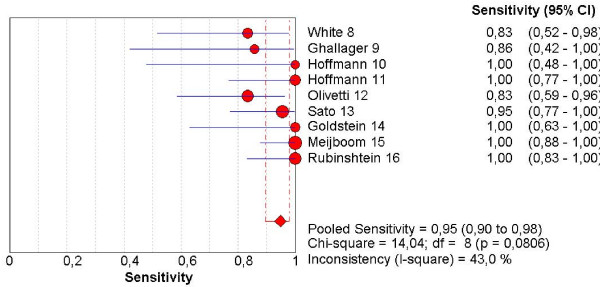
Forest plot of sensitivity on a per patient basis.

**Figure 5:**
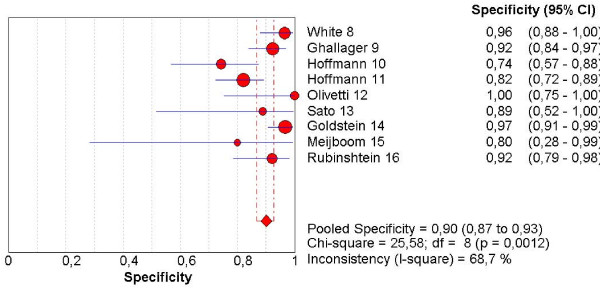
Forest plot of specificity on a per patient basis.

**Figure 6:**
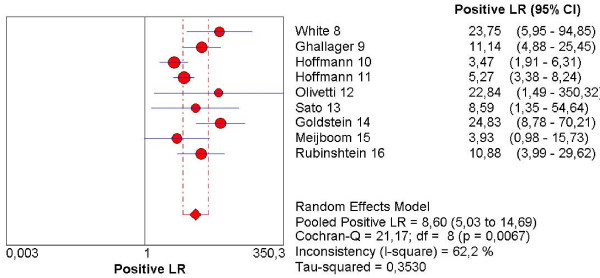
Forest plot of positive likelihood ratio on a per patient basis. LR: Likelihood ratio.

**Figure 7:**
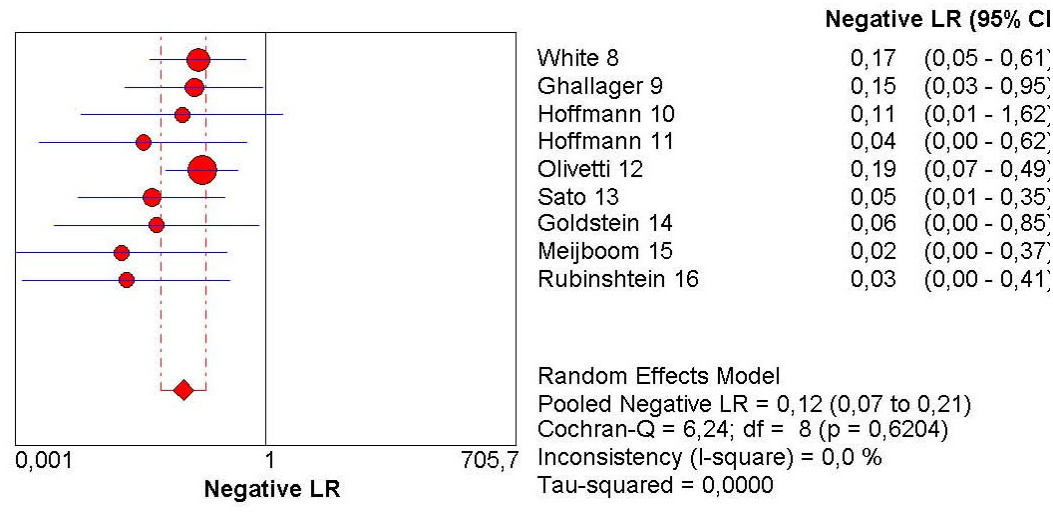
Forest plot of negative likelihood ratio on a per patient basis. LR: Likelihood ratio.

**Figure 8:**
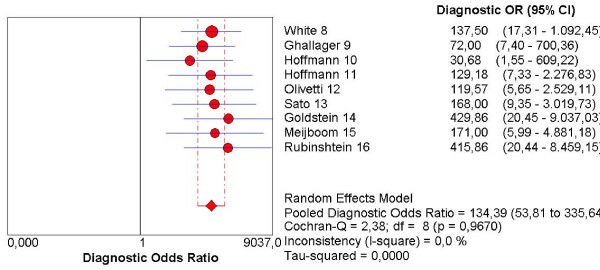
Forest plot of diagnostic odds ratio on a per patient basis. OR: Odds ratio.

The pooled sensitivity, specificity, NLR, PLR and the DOR are graphically displayed as forest plots in Fig. 4, 5, 6, 7, 8 and show the following values: Pooled DOR was 134.39 (95%CI, 53.81–335.64). The pooled sensitivity and specificity were 0.95 (95%CI, 0.90–0.98) and 0.90 (95%CI, 0.87–0.93). The pooled NLR and PLR were 0.12 (95%CI, 0.06–0.21) and 8.60 (95%CI, 5.03–14.70).

**Figure 9:**
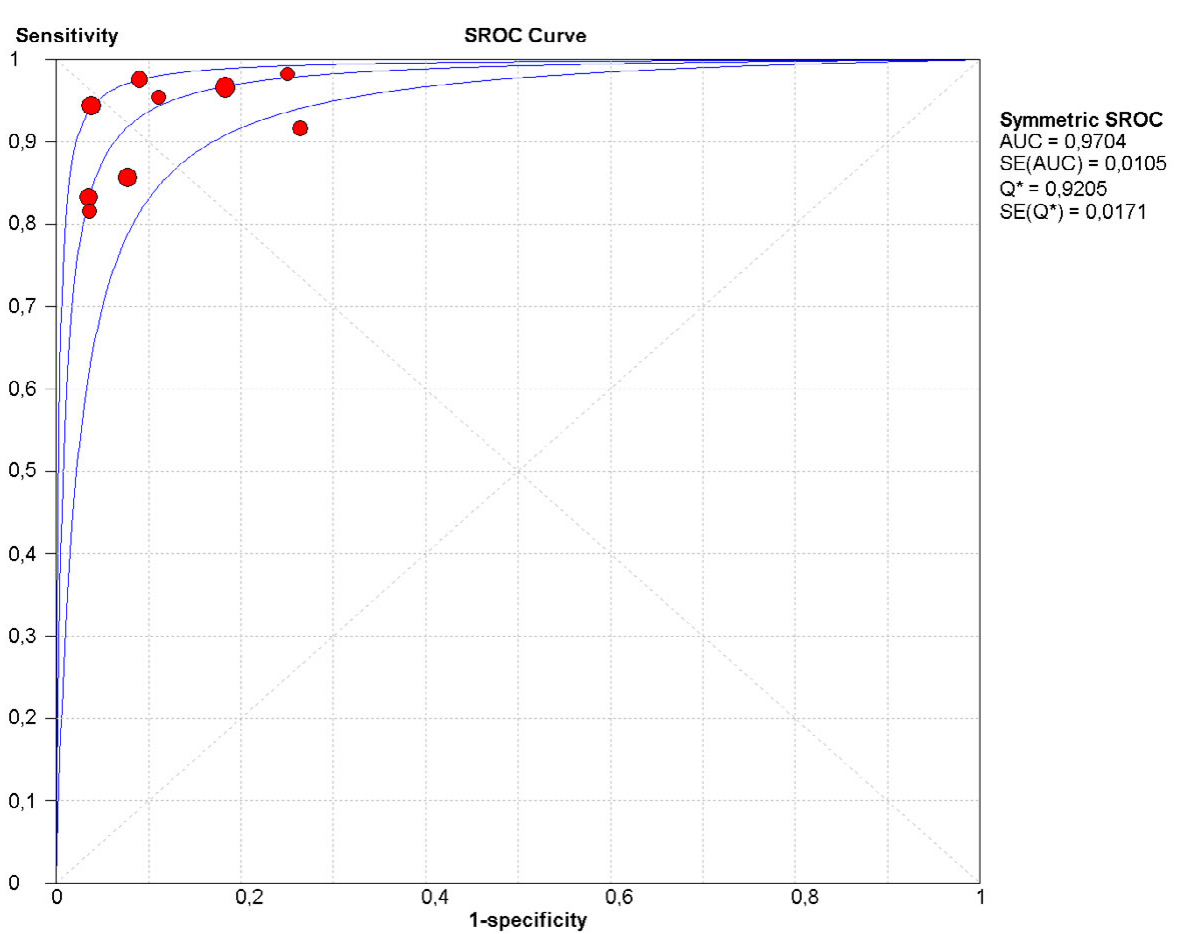
SROC curve of per patient analysis. AUC: Area under the curve. SE: Standard error. Q*: Point of intersection of the SROC curve where SE and SP are equal.

SROC plot is found in Figure 9 and displays an excellent value for AUC (0.97, SE 0.01) and Q* (0.92, SE 0.01).

Pooled NAP was 0.03 (95%CI, 0.01 to 0.07)

The results of the logistic (meta-)regressions showed that none of the investigated variables had influence on the diagnostic performance or NAP.

## Discussion

Our analysis of the available literature on MDCTA for the diagnosis of NSTEMI and/or UAP revealed that the overall pooled estimates of sensitivity and specificity on a patient-level were good to excellent. Furthermore, the pooled estimates of NLR and PLR (0.12 and 8.60 repectively) and the results of the logistic (meta-) regressions revealed that the test can be used as a very reliable imaging modality to rule out NSTEMI and UAP in an acute setting, irrespective of scanner hardware and certain patient characteristics such as age or gender. The number of non-diagnostic scans was as low as 3%, which showed that the technique seems to be robust. Moreover, detailed statistical analysis showed that this meta-analysis did not suffer from frequently occurring problems such as publication bias or extreme heterogeneity/inconsistency between studies.

A previous meta-analysis [34] that studied MDCTA in comparison with CA in a non-acute setting revealed important heterogeneity and some publication bias, especially when the analysis was done on a per segment basis, and demonstrated a prominent influence of the number of detectors on the number of non-assessable segments and diagnostic performance. Also the exclusion of segments before analysis had an important influence on diagnostic performance. The reason that these effects are statistically not demonstrable in the current study is probably due to the rather small sample and to the type of analysis, that was done exclusively on a per patient level.

This is an important observation: MDCTA can probably be used as a reliable technique to rule out NSTEMI or UAP in a acute setting and the test can be done very quickly and does not require complex patient preparation or logistic resources. The scan can be performed in the first hours after the onset of the clinical complaints and when negative, the patient can be discharged from the emergency department immediately. In the studies that were pooled in this meta-analysis, there was one randomized study that prooved that the average time from randomization to diagnosis was significantly shorter in the MDCTA arm than in the other arm (3.4 h versus 15 h)[14].

Although the purpose of this study was not to formally study the reliability of MDCTA to exclude all causes of chest pain, it is apparent from the individual data from most studies in this series that alternative diagnoses besides coronary disease can often be ruled out.

Other imaging techniques have been used to develop a quick and efficient triage strategy for acute chest pain in the emergency department. Nuclear medicine techniques and to a much lesser degree, echocardiography, have been extensively tested, and there is a large body of literature supporting its use in these circumstances [35]. In 2003 a joint task force of the American College of Cardiology, the American Heart Association and the American Society for Nuclear Cardiology published guidelines for the use of Myocardial perfusion imaging (MPI) [36]. The task force gave a class 1 recommendation to the use of acute rest MPI for the assessment of patients presenting with a possible acute coronary syndrome in whom initial markers and the ECG are non diagnostic. A randomized study [37] showed that MPI improves triage and that unnecessary hospitalisation was reduced among patients without acute ischaemia, without reducing appropriate admission for patients with acute ischemia. Wether MDCTA can be recommended as a substitute for MPI needs to be investigated further, since the total number of patients in this study is low.

Radiation dose may be a matter of debate. A clear disadvantage of MDCTA is the relatively high radiation dose that goes with the examination, with average doses ranging from 10 to 20 mS [38]. How this relates to the dose of MPI now and in the future is a matter that goes beyond this discussion, but it may be anticipated that radiation dose reducing protocols for MDCTA will have an important impact [38].

Recently a few studies have described the use of three dimensional whole heart MRI for imaging the coronary tree in unstable patients. This technique has two advantages over MDCTA namely the lack of ionising radiation and no need to inject iodine containing contrast agents [39,40].

Some limitations of this study have to be acknowledged. We put the presence of obstructive coronary disease and positive biomarkers on a similar level as an outcome of positive diagnosis. This is clearly an oversimplification and would suggest that every patient with obstructive disease has an ACS. This is obviously not the case. In the logistic meta-regression, there was however no influence of the type of outcome used on the diagnostic performance.

The number of patients that was pooled in this analysis is rather low and the conclusions may be misleading to some degree. However, one of the studies incorporated was a randomized trial, and the conclusions drawn from this individual study were in line with the global conclusion, which is supporting the suggestion that the pooled conclusions are probably realistic [14]. Another, probably more important limitation is that detailed information on factors that technically limit MDCTA, such as high calcium score, irregular rythm, the presence of stents and dyspnea [41-44] was not mentioned in most studies, or at least not amenable to pooling. The same was true for pre-test probability of the patient populations. This may cast some doubt on the potential to generalize the results to all patient populations.

The question may be asked why we did a meta-analysis on acute patients, since meta-analysis of MDCTA of the coronary arteries has already been performed previously [34]. We considered the studies in the acute setting as a different and interesting subgroup for two reasons. First, the studies that were included looked at outcome in a broader sense: clinical and/or conventional angiography. None of the studies included here has been included in this previous meta-analysis. Second the acute setting was supposed to include patients that may have a more dramatic clinical presentation in which MDCTA might result in more non-diagnostic scans. This selection resulted, as quoted already, in a small pooled group, with all attendant draw-backs.

## Conclusion

The conclusion of this study is that MDCTA may be a safe and quick diagnostic technique to rule out NSTEMI or UAP in the emergency department. Future randomized studies should focus on including subtypes of patients that are known to be more difficult to image with MDCTA or to determine what the precise indication is for MDCTA and the place in the diagnostic algorithm. Cost-effectiveness studies should be done to investigate the economic impact of using this technique to evaluate chest pain in an acute setting.

## Competing interests

The author(s) declare that they have no competing interests.

## Authors' contributions

PV carried out the systematic search, statistical analysis, guarantor of study integrity, manuscript authoring and submission, data collection and prepared the manuscript. ID carried out the statistical analysis and contributed to the manuscript writing. OB, GS, CB and WW provided clinical advice and commented on the manuscript. All authors read and approved the final manuscript.

## Pre-publication history

The pre-publication history for this paper can be accessed here:



## Supplementary Material

Additional file 1 Search strategies. the data provided illustrate the search strategy followed Click here for file

Additional file 2 Reasons for excluding articles. a list of excluded articles grouped in categories is given. Click here for file
