# Supplementary material for: Detection of non-ST-elevation myocardial infarction and unstable angina in the acute setting: meta-analysis of diagnostic performance of multi-detector computed tomographic angiography
Source: BMC Cardiovasc Disord. 2007 Dec 19;7:39. doi: 10.1186/1471-2261-7-39 (PMC2228319; doi:10.1186/1471-2261-7-39)
Supplement: Additional file 1 — Search strategies. the data provided illustrate the search strategy followed [file 1471-2261-7-39-S1.doc]

**Search strategies**

a)Search strategy 1 (16)

#1 Search(("acute coronary syndrome"[MeSH Terms] OR chest pain[Text Word]) OR ("coronary angiography"[MeSH Terms] OR coronary angiography[Text Word])) AND ("tomography, spiral computed"[MeSH Terms] OR spiral computed tomography[Text Word]) AND (("sensitivity andspecificity" [TIAB] NOT Medline[SB]) OR "sensitivity and specificity" [MeSH Terms] OR sensitivity [Text Word])

#2 Search(((((((((("sensitivity and specificity" [All Fields] OR "sensitivity and specificity/standards"[All Fields]) OR "specificity"[All Fields]) OR "screening" [All Fields]) OR "false positive"[All Fields]) OR "false negative"[All Fields]) OR "accuracy"[All Fields]) OR (((("predictive value"[All Fields] OR "predictive value of tests"[All Fields]) OR "predictive value of tests/standards" [All Fields]) OR "predictive values" [All Fields]) OR "predictive values of tests" [All Fields])) OR (("reference value" [All Fields] OR "reference values"[All Fields]) OR "reference values/standards" [All Fields])) OR ((((((((((("roc" [All Fields] OR "roc analyses" [All Fields]) OR "roc analysis"[All Fields]) OR "roc and"[All Fields]) OR "roc area"[All Fields]) OR "roc auc"[All Fields]) OR "roc characteristics" [All Fields]) OR "roc curve"[All Fields]) OR "roc curve method"[All Fields]) OR "roccurves" [All Fields]) OR "roc estimated" [All Fields]) OR "roc evaluation" [All Fields])) OR "likelihood ratio"[All Fields]) AND ("coronary vessels" [MeSH Terms] OR Coronary Vessels[Text Word])AND computed tomography

#3 Search SENSITIVITY OR SPECIFICITY OR CORONARY

#4 Search COMPUTED TOMOGRAPHY

#5 Search #3 AND #4

#6 Search #5 AND “coronary arteries”

#7 Search #6 OR #2 OR#1

b) Search strategy 2 (17)

|  [#19 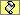](javascript:PopUpMenu2_Set(QMenu19);) | Search **#18 AND #12 AND #13** |
| --- | --- |
| [#18 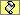](javascript:PopUpMenu2_Set(QMenu18);) | Search **chest AND pain** |
| [#17 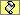](javascript:PopUpMenu2_Set(QMenu17);) | Search **#11 AND #12 AND #13** |
| [#16 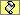](javascript:PopUpMenu2_Set(QMenu16);) | Search **#1 AND #6** |
| [#15 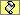](javascript:PopUpMenu2_Set(QMenu15);) | Search **# 8 AND # 11 AND # 12 AND #13 AND #14** |
| [#14 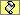](javascript:PopUpMenu2_Set(QMenu14);) | Search **#10 OR #9** |
| [#13 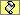](javascript:PopUpMenu2_Set(QMenu13);) | Search **#6 OR #7** |
| [#12 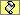](javascript:PopUpMenu2_Set(QMenu12);) | Search **#5 OR #4** |
| [#11 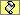](javascript:PopUpMenu2_Set(QMenu11);) | Search **#1 OR #2 OR #3** |
| [#10 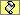](javascript:PopUpMenu2_Set(QMenu10);) | Search **angiography** |
| [#9 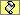](javascript:PopUpMenu2_Set(QMenu9);) | Search **coronary angiography** |
| [#8 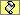](javascript:PopUpMenu2_Set(QMenu8);) | Search **diagnosis** |
| [#7 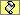](javascript:PopUpMenu2_Set(QMenu7);) | Search **tomography, x-ray computed** |
| [#6 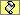](javascript:PopUpMenu2_Set(QMenu6);) | Search **tomography, spiral computed** |
| [#5 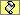](javascript:PopUpMenu2_Set(QMenu5);) | Search **emergency** |
| [#4 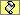](javascript:PopUpMenu2_Set(QMenu4);) | Search **acute** |
| [#3 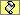](javascript:PopUpMenu2_Set(QMenu3);) | Search **coronary vessels** |
| [#2 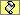](javascript:PopUpMenu2_Set(QMenu2);) | Search **angina pectoris, variant** |
| [#1 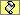](javascript:PopUpMenu2_Set(QMenu1);) | Search **angina, unstable** |
