# Supplementary material for: Detection of non-ST-elevation myocardial infarction and unstable angina in the acute setting: meta-analysis of diagnostic performance of multi-detector computed tomographic angiography
Source: BMC Cardiovasc Disord. 2007 Dec 19;7:39. doi: 10.1186/1471-2261-7-39 (PMC2228319; doi:10.1186/1471-2261-7-39)
Supplement: Additional file 2 — Reasons for excluding articles. a list of excluded articles grouped in categories is given. [file 1471-2261-7-39-S2.doc]

# Reasons for excluding articles

# Not an acute ACS setting

1. Achenbach S, Giesler T, Ropers D et al. Detection of coronary artery stenoses by contrast-enhanced, retrospectively electrocardiographically-gated, multislice spiral computed tomography. Circulation 2001;103:2535-8.

2. Achenbach S, Ropers D, Pohle K et al. Clinical results of minimally invasive coronary angiography using computed tomography. Cardiol Clin 2003;21:549-59.

3. Achenbach S, Ropers D, Pohle FK et al. Detection of coronary artery stenoses using multi-detector CT with 16 x 0.75 collimation and 375 ms rotation. Eur Heart J 2005;26:1978-86.

4. Aviram G, Finkelstein A, Herz I et al. Clinical value of 16-slice multi-detector CT compared to invasive coronary angiography. Int J Cardiovasc Intervent 2005;7:21-8.

5. Becker CR, Knez A, Leber A et al. Detection of coronary artery stenoses with multislice helical CT angiography. J Comput Assist Tomogr 2002;26:750-5.

6. Blinder G, Benhorin J, Koukoui D, Zimam R, Hiller N. The value of electrocardiography-gated multi-slice computed tomography in the evaluation of patients with chest pain. Isr Med Assoc J 2005;7:419-23.

7. Bonmassari R, Muraglia S, Centonze M, Coser D, Stoppa G, Disertori M. Noninvasive detection of coronary artery stenosis with 16-slice spiral computed tomography in a population at low to moderate risk for coronary artery disease. J Cardiovasc Med (Hagerstown ) 2006;7:817-25.

8. Burgstahler C, Beck T, Kuettner A et al. Image quality and diagnostic accuracy of 16-slice multidetector computed tomography for the detection of coronary artery disease in obese patients. Int J Obes (Lond) 2006;30:569-73.

9. Cademartiri F, Nieman K, Mollet N et al. [Non-invasive 16-row spiral multislice computed tomography coronary angiography after one year of experience]. Ital Heart J Suppl 2003;4:587-93.

10. Cademartiri F, Runza G, Marano R et al. Diagnostic accuracy of 16-row multislice CT angiography in the evaluation of coronary segments. Radiol Med (Torino) 2005;109:91-7.

11. Cordeiro MA, Miller JM, Schmidt A et al. Non-invasive half millimetre 32 detector row computed tomography angiography accurately excludes significant stenoses in patients with advanced coronary artery disease and high calcium scores. Heart 2006;92:589-97.

12. Ehara M, Surmely JF, Kawai M et al. Diagnostic accuracy of 64-slice computed tomography for detecting angiographically significant coronary artery stenosis in an unselected consecutive patient population: comparison with conventional invasive angiography. Circ J 2006;70:564-71.

13. Erdogan N, Akar N, Vural M et al. Diagnostic value of 16-slice multidetector computed tomography in symptomatic patients with suspected significant obstructive coronary artery disease. Heart Vessels 2006;21:278-84.

14. Fine JJ, Hopkins CB, Hall PA, Delphia RE, Attebery TW, Newton FC. Noninvasive coronary angiography: agreement of multi-slice spiral computed tomography and selective catheter angiography. Int J Cardiovasc Imaging 2004;20:549-52.

15. Fine JJ, Hopkins CB, Ruff N, Newton FC. Comparison of accuracy of 64-slice cardiovascular computed tomography with coronary angiography in patients with suspected coronary artery disease. Am J Cardiol 2006;97:173-4.

16. Francone M, Carbone I, Danti M et al. ECG-gated multi-detector row spiral CT in the assessment of myocardial infarction: correlation with non-invasive angiographic findings. Eur Radiol 2006;16:15-24.

17. Garcia MJ, Lessick J, Hoffmann MH. Accuracy of 16-row multidetector computed tomography for the assessment of coronary artery stenosis. JAMA 2006;296:403-11.

18. Gaspar T, Dvir D, Peled N. The role of 16-slice computed tomography angiography in the diagnosis of coronary artery disease: large sample analysis. Isr Med Assoc J 2005;7:424-7.

19. Gaudio C, Mirabelli F, Alessandra L et al. Noninvasive assessment of coronary artery stenoses by multidetector-row spiral computed tomography: comparison with conventional angiography. Eur Rev Med Pharmacol Sci 2005;9:13-21.

20. Gerber TC, Kuzo RS, Karstaedt N et al. Current results and new developments of coronary angiography with use of contrast-enhanced computed tomography of the heart. Mayo Clin Proc 2002;77:55-71.

21. Giesler T, Baum U, Ropers D et al. Noninvasive visualization of coronary arteries using contrast-enhanced multidetector CT: influence of heart rate on image quality and stenosis detection. AJR Am J Roentgenol 2002;179:911-6.

22. Grosse C, Globits S, Hergan K. Forty-slice spiral computed tomography of the coronary arteries: assessment of image quality and diagnostic accuracy in a non-selected patient population. Acta Radiol 2007;48:36-44.

23. Gulati GS, Seth S, Kurian S, Jagia P, Sharma S. Non-invasive diagnosis of coronary artery disease with 16-slice computed tomography. Natl Med J India 2005;18:236-41.

24. Halon DA, Gaspar T, Adawi S et al. Uses and Limitations of 40 Slice Multi-Detector Row Spiral Computed Tomography for Diagnosing Coronary Lesions in Unselected Patients Referred for Routine Invasive Coronary Angiography. Cardiology 2006;108:200-9.

25. Herzog C, Britten M, Balzer JO et al. Multidetector-row cardiac CT: diagnostic value of calcium scoring and CT coronary angiography in patients with symptomatic, but atypical, chest pain. Eur Radiol 2004;14:169-77.

26. Heuschmid M, Kuettner A, Schroeder S et al. ECG-gated 16-MDCT of the coronary arteries: assessment of image quality and accuracy in detecting stenoses. AJR Am J Roentgenol 2005;184:1413-9.

27. Hoffmann U, Moselewski F, Cury RC et al. Predictive value of 16-slice multidetector spiral computed tomography to detect significant obstructive coronary artery disease in patients at high risk for coronary artery disease: patient-versus segment-based analysis. Circulation 2004;110:2638-43.

28. Holmstrom M, Vesterinen P, Hanninen H, Sillanpaa MA, Kivisto S, Lauerma K. Noninvasive analysis of coronary artery disease with combination of MDCT and functional MRI. Acad Radiol 2006;13:177-85.

29. Kaiser C, Bremerich J, Haller S et al. Limited diagnostic yield of non-invasive coronary angiography by 16-slice multi-detector spiral computed tomography in routine patients referred for evaluation of coronary artery disease. Eur Heart J 2005;26:1987-92.

30. Kaufmann PA. Accuracy of noninvasive coronary angiography using computed tomography. J Am Coll Cardiol 2006;48:219.

31. Kefer J, Coche E, Legros G et al. Head-to-head comparison of three-dimensional navigator-gated magnetic resonance imaging and 16-slice computed tomography to detect coronary artery stenosis in patients. J Am Coll Cardiol 2005;46:92-100.

32. Kopp AF, Ohnesorge B, Flohr T et al. [Cardiac multidetector-row CT: first clinical results of retrospectively ECG-gated spiral with optimized temporal and spatial resolution]. Rofo 2000;172:429-35.

33. Kopp AF, Schroeder S, Kuettner A et al. Non-invasive coronary angiography with high resolution multidetector-row computed tomography. Results in 102 patients. Eur Heart J 2002;23:1714-25.

34. Kuettner A, Trabold T, Schroeder S et al. Noninvasive detection of coronary lesions using 16-detector multislice spiral computed tomography technology: initial clinical results. J Am Coll Cardiol 2004;44:1230-7.

35. Kuettner A, Kopp AF, Schroeder S et al. Diagnostic accuracy of multidetector computed tomography coronary angiography in patients with angiographically proven coronary artery disease. J Am Coll Cardiol 2004;43:831-9.

36. Kuettner A, Beck T, Drosch T et al. Image quality and diagnostic accuracy of non-invasive coronary imaging with 16 detector slice spiral computed tomography with 188 ms temporal resolution. Heart 2005;91:938-41.

37. Kuettner A, Beck T, Drosch T et al. Diagnostic accuracy of noninvasive coronary imaging using 16-detector slice spiral computed tomography with 188 ms temporal resolution. J Am Coll Cardiol 2005;45:123-7.

38. Lau GT, Ridley LJ, Schieb MC et al. Coronary artery stenoses: detection with calcium scoring, CT angiography, and both methods combined. Radiology 2005;235:415-22.

39. Leber AW, Knez A, von Ziegler F et al. Quantification of obstructive and nonobstructive coronary lesions by 64-slice computed tomography: a comparative study with quantitative coronary angiography and intravascular ultrasound. J Am Coll Cardiol 2005;46:147-54.

40. Leschka S, Alkadhi H, Plass A et al. Accuracy of MSCT coronary angiography with 64-slice technology: first experience. Eur Heart J 2005;26:1482-7.

41. Lim MC, Wong TW, Yaneza LO, De Larrazabal C, Lau JK, Boey HK. Non-invasive detection of significant coronary artery disease with multi-section computed tomography angiography in patients with suspected coronary artery disease. Clin Radiol 2006;61:174-80.

42. Martuscelli E, Romagnoli A, D'Eliseo A et al. Accuracy of thin-slice computed tomography in the detection of coronary stenoses. Eur Heart J 2004;25:1043-8.

43. Mollet NR, Cademartiri F, Van Mieghem CA et al. High-resolution spiral computed tomography coronary angiography in patients referred for diagnostic conventional coronary angiography. Circulation 2005;112:2318-23.

44. Mollet NR, Cademartiri F, Krestin GP et al. Improved diagnostic accuracy with 16-row multi-slice computed tomography coronary angiography. J Am Coll Cardiol 2005;45:128-32.

45. Moon JY, Chung N, Choi BW et al. The utility of multi-detector row spiral CT for detection of coronary artery stenoses. Yonsei Med J 2005;46:86-94.

46. Morgan-Hughes GJ, Marshall AJ, Roobottom CA. Multislice computed tomographic coronary angiography: experience in a UK centre. Clin Radiol 2003;58:378-83.

47. Morgan-Hughes GJ, Roobottom CA, Owens PE, Marshall AJ. Highly accurate coronary angiography with submillimetre, 16 slice computed tomography. Heart 2005;91:308-13.

48. Nieman K, Rensing BJ, Van Geuns RJ et al. Non-invasive coronary angiography with multislice spiral computed tomography: impact of heart rate. Heart 2002;88:470-4.

49. Nieman K, Cademartiri F, Lemos PA, Raaijmakers R, Pattynama PM, de Feyter PJ. Reliable noninvasive coronary angiography with fast submillimeter multislice spiral computed tomography. Circulation 2002;106:2051-4.

50. Nieman K, Rensing BJ, Van Geuns RJ et al. Usefulness of multislice computed tomography for detecting obstructive coronary artery disease. Am J Cardiol 2002;89:913-8.

51. Nikolaou K, Knez A, Rist C et al. Accuracy of 64-MDCT in the diagnosis of ischemic heart disease. AJR Am J Roentgenol 2006;187:111-7.

52. Nikolaou K, Rist C, Wintersperger BJ et al. Clinical value of MDCT in the diagnosis of coronary artery disease in patients with a low pretest likelihood of significant disease. AJR Am J Roentgenol 2006;186:1659-68.

53. Ong K, Chin SP, Chan WL et al. Feasibility and accuracy of 64-row MDCT coronary imaging from a centre with early experience: a review and comparison with established centres. Med J Malaysia 2005;60:629-36.

54. Ong TK, Chin SP, Liew CK et al. Accuracy of 64-row multidetector computed tomography in detecting coronary artery disease in 134 symptomatic patients: influence of calcification. Am Heart J 2006;151:1323-6.

55. Paul JF, Ohanessian A, Caussin C et al. [Visualization of coronary tree and detection of coronary artery stenosis using 16-slice, sub-millimeter computed tomography: preliminary experience]. Arch Mal Coeur Vaiss 2004;97:31-6.

56. Probst C, Kovacs A, Schmitz C, Schiller W, Schild H, Welz A. Quantification of coronary artery stenosis with 16-slice MSCT in patients before CABG surgery: comparison to standard invasive coronary angiography. Heart Surg Forum 2005;8:E42-E46.

57. Raff GL, Gallagher MJ, O'Neill WW, Goldstein JA. Diagnostic accuracy of noninvasive coronary angiography using 64-slice spiral computed tomography. J Am Coll Cardiol 2005;46:552-7.

58. Rodevand O, Hogalmen G, Gudim LP, Indrebo T, Molstad P, Vandvik PO. Limited usefulness of non-invasive coronary angiography with 16-detector multislice computer tomography at a community hospital. Scand Cardiovasc J 2006;40:76-82.

59. Ropers D, Rixe J, Anders K et al. Usefulness of multidetector row spiral computed tomography with 64- x 0.6-mm collimation and 330-ms rotation for the noninvasive detection of significant coronary artery stenoses. Am J Cardiol 2006;97:343-8.

60. Sato Y, Matsumoto N, Kato M et al. Noninvasive assessment of coronary artery disease by multislice spiral computed tomography using a new retrospectively ECG-gated image reconstruction technique. Circ J 2003;67:401-5.

61. Scheffel H, Alkadhi H, Plass A et al. Accuracy of dual-source CT coronary angiography: First experience in a high pre-test probability population without heart rate control. Eur Radiol 2006;16:2739-47.

62. Schlosser T, Konorza T, Hunold P, Kuhl H, Schmermund A, Barkhausen J. Noninvasive visualization of coronary artery bypass grafts using 16-detector row computed tomography. J Am Coll Cardiol 2004;44:1224-9.

63. Schmermund A, Baumgart D, Sack S et al. Assessment of coronary calcification by electron-beam computed tomography in symptomatic patients with normal, abnormal or equivocal exercise stress test. Eur Heart J 2000;21:1674-82.

64. Schroeder S, Kopp AF, Kuettner A et al. Influence of heart rate on vessel visibility in noninvasive coronary angiography using new multislice computed tomography: experience in 94 patients. Clin Imaging 2002;26:106-11.

65. Schuijf JD, Bax JJ, Jukema JW et al. Noninvasive angiography and assessment of left ventricular function using multislice computed tomography in patients with type 2 diabetes. Diabetes Care 2004;27:2905-10.

66. Schuijf JD, Bax JJ, Salm LP et al. Noninvasive coronary imaging and assessment of left ventricular function using 16-slice computed tomography. Am J Cardiol 2005;95:571-4.

67. Schuijf JD, Bax JJ, Jukema JW et al. Noninvasive evaluation of the coronary arteries with multislice computed tomography in hypertensive patients. Hypertension 2005;45:227-32.

68. Schuijf JD, Pundziute G, Jukema JW et al. Diagnostic accuracy of 64-slice multislice computed tomography in the noninvasive evaluation of significant coronary artery disease. Am J Cardiol 2006;98:145-8.

69. Treede H, Becker C, Reichenspurner H et al. Multidetector computed tomography (MDCT) in coronary surgery: first experiences with a new tool for diagnosis of coronary artery disease. Ann Thorac Surg 2002;74:S1398-S1402.

70. Vogl TJ, Abolmaali ND, Diebold T et al. Techniques for the detection of coronary atherosclerosis: multi-detector row CT coronary angiography. Radiology 2002;223:212-20.

1. Watkins MW, Hesse B, Green CE et al. Detection of coronary artery stenosis using 40-channel computed tomography with multi-segment reconstruction. Am J Cardiol 2007;99:175-81.

# Unknown status of/or positive biomarkers

1. Coles DR, Wilde P, Oberhoff M, Rogers CA, Karsch KR, Baumbach A. Multislice computed tomography coronary angiography in patients admitted with a suspected acute coronary syndrome
1. Int J Cardiovasc Imaging 2006.

2. Dirksen MS, Jukema JW, Bax JJ et al. Cardiac multidetector-row computed tomography in patients with unstable angina. Am J Cardiol 2005;95:457-61.

3. Dorgelo J, Willems TP, Geluk CA, van Ooijen PM, Zijlstra F, Oudkerk M. Multidetector computed tomography-guided treatment strategy in patients with non-ST elevation acute coronary syndromes: a pilot study. Eur Radiol 2005;15:708-13.

4. Ghersin E, Litmanovich D, Dragu R et al. 16-MDCT coronary angiography versus invasive coronary angiography in acute chest pain syndrome: a blinded prospective study. AJR Am J Roentgenol 2006;186:177-84.

5. Johnson TR, Nikolaou K, Wintersperger BJ et al. ECG-gated 64-MDCT angiography in the differential diagnosis of acute chest pain. AJR Am J Roentgenol 2007;188:76-82.

6. Meijboom WB, Mollet NR, van Mieghem CA et al. 64-slice Computed Tomography Coronary Angiography in Patients with Non-ST Elevation Acute Coronary Syndrome. Heart 2007.

7. Schroeder S, Kuettner A, Beck T et al. Usefulness of noninvasive MSCT coronary angiography as first-line imaging technique in patients with chest pain: initial clinical experience. Int J Cardiol 2005;102:469-75.

# Review article

1. Achenbach S. Computed tomography coronary angiography. J Am Coll Cardiol 2006;48:1919-28.

2. de Feyter P, Mollet N, Nieman K et al. Noninvasive visualisation of coronary atherosclerosis with multislice computed tomography. Cardiovasc Radiat Med 2004;5:49-56.

3. Hoffmann MH, Shi H, Schmitz BL et al. Noninvasive coronary angiography with multislice computed tomography. JAMA 2005;293:2471-8.

4. Hoffmann MH, Lessick J. Multidetector-row computed tomography for noninvasive coronary imaging. Expert Rev Cardiovasc Ther 2006;4:583-94.

5. Schuijf JD, Mollet NR, Cademartiri F et al. Do risk factors influence the diagnostic accuracy of noninvasive coronary angiography with multislice computed tomography? J Nucl Cardiol 2006;13:635-41.

6. White C, Read K, Kuo D. Assessment of chest pain in the emergency room: what is the role of multidetector CT? Eur J Radiol 2006;57:368-72.

# No comparison with reference standard in all cases

1. Savino G, Herzog C, Costello P, Schoepf UJ. 64 slice cardiovascular CT in the emergency department: concepts and first experiences. Radiol Med (Torino) 2006;111:481-96.
